# Pseudotrypsin: A Little-Known Trypsin Proteoform

**DOI:** 10.3390/molecules23102637

**Published:** 2018-10-14

**Authors:** Zdeněk Perutka, Marek Šebela

**Affiliations:** Department of Protein Biochemistry and Proteomics, Centre of the Region Haná for Biotechnological and Agricultural Research, Faculty of Science, Palacký University, Šlechtitelů 27, 783 71 Olomouc, Czech Republic; Perutka.Zdenek@seznam.cz

**Keywords:** autolysis, chain, cleavage, digestion, peptide, proteoform, pseudotrypsin, specificity, trypsin

## Abstract

Trypsin is the protease of choice for protein sample digestion in proteomics. The most typical active forms are the single-chain β-trypsin and the two-chain α-trypsin, which is produced by a limited autolysis of β-trypsin. An additional intra-chain split leads to pseudotrypsin (ψ-trypsin) with three chains interconnected by disulfide bonds, which can be isolated from the autolyzate by ion-exchange chromatography. Based on experimental data with artificial substrates, peptides, and protein standards, ψ-trypsin shows altered kinetic properties, thermodynamic stability and cleavage site preference (and partly also cleavage specificity) compared to the above-mentioned proteoforms. In our laboratory, we have analyzed the performance of bovine ψ-trypsin in the digestion of protein samples with a different complexity. It cleaves predominantly at the characteristic trypsin cleavage sites. However, in a comparison with common tryptic digestion, non-specific cleavages occur more frequently (mostly after the aromatic residues of Tyr and Phe) and more missed cleavages are generated. Because of the preferential cleavages after the basic residues and more developed side specificity, which is not expected to occur for the major trypsin forms (but may appear anyway because of their autolysis), ψ-trypsin produces valuable information, which is complementary in part to data based on a strictly specific trypsin digestion and thus can be unnoticed following common proteomics protocols.

## 1. Cleavage Specificity of Trypsin

Trypsin, a serine protease, is commonly used as an important enzymatic reagent in biochemistry and biology. It is almost indispensable especially for the digestion of protein samples to peptides in bottom-up proteomics [1]. Apart from this application, trypsin is a tool in working with cell cultures. During trypsinization, surface adhesion proteins are degraded, which allows adherent cells to be detached from each other and the walls of plastic containers or plates in which they are being cultured. In industry, interestingly, trypsin is applied to hydrolyze allergenic proteins for the production of hypoallergenic milk [2]. In proteomics, sample digests are typically analyzed for protein identification by nanoflow liquid chromatography coupled to tandem mass spectrometry (nLC-MS/MS). MS-based data on peptides are searched against amino acid sequence databases, which benefits from the relative stringent cleavage specificity of trypsin as the search algorithms incorporate the cleavage rule as a filtering criterion. According to a study with complex samples from 2004, the enzyme cleaves peptide bonds in proteins at pH 8.3 exclusively at the carboxyl end of arginine and lysine residues [3]. This is in agreement with the canonical trypsin cleavage rule postulated a long time ago [4] even though it was built on results obtained at that time only with a limited amount of substrates [5]. Thus, it has long been accepted that trypsin does not cleave before proline and its activity is suppressed either if a cysteine appears next to Arg/Lys or the basic residue is N- or C-terminally adjacent to an acidic residue. This old rule was questioned when a large data set of 14.5 million MS/MS spectra of peptides from *Shewanella oneidensis* was processed to statistically evaluate the cleavage sites [5]. Interestingly, numerous cleavages before proline were found. Their number was even higher than that referring to the cleavages before cysteine.

An average length of tryptic peptides is 14 amino acids. This number has been deduced from an in silico digestion of human proteins in the UniProt database [1]. Because of this reasonable size as well as the presence of a positive charge at the C-terminal Arg or Lys, which enhances the ionization process in the positive ionization mode, tryptic peptides are highly amenable to mass spectrometric measurements. In fact, there are at least two defined positive charges in tryptic peptides (at both N- and C-termini), which is favorable for a good fragmentation in MS/MS analyses [1,2].

## 2. Nonspecific and Missed Cleavage Sites

In addition to the cleavages after Arg or Lys, proteomics studies have often reported the formation of semitryptic and nonspecific peptides during the digestion process involving trypsin [1,6]. The semitryptic cleavage assumes that one of the cleavage sites is tryptic, but the other site may be at any residue. A minor chymotrypsin or chymotrypsin-like activity yields nonspecific cleavages C-terminal to phenylalanine, tyrosine, tryptophan, or leucine residues [7]. This can result either from the presence of a chymotrypsin contamination, which is variable in trypsin preparations supplied by different vendors [1], or pseudotrypsin (ψ-trypsin), a product of trypsin autolysis, which possesses such an activity in addition to the characteristic trypsin properties [8]. The presence of nonspecific peptides in tryptic digests (excluding C-terminal peptides) is also elucidated by a secondary non-enzymatic cleavage between Asp and Pro residues yielding peptides with an N-terminal proline. This is because of the lability of the respective bond, which is easily hydrolyzed in solution as well as broken in the gas phase [3].

When the protein substrate is not cleaved to a completion, missed cleavages occur, which make the assignment of experimental data to amino acid sequence databases less specific and straightforward. Missed cleavage sites were investigated for example by matrix-assisted laser desorption/ionization time-of-flight mass spectrometry (MALDI-TOF MS) using human cell line or *Mycobacterium* proteins separated by two-dimensional gel electrophoresis and digested in-gel by a porcine trypsin [9]. The analysis showed that about 90 % of the detected peptides with missed cleavage sites could be attributed to the following sequence motifs: (1) Arg or Lys with a neighboring proline at the C-terminal side, (2) two successive basic residues (Arg-Arg, Arg-Lys, Lys-Arg, Lys-Lys), and (3) Arg or Lys with an aspartic acid or glutamic acid residue at either N-terminal or C-terminal side. When processing peptide MS- and MS/MS-based data by database searches (during which theoretical peptide sequences are generated according to the selected input cleavage rules), the user may, among others, adjust a maximum number of missed cleavage sites. Usually, a setting of 0, 1, or 2 missed cleavages for peptides is recommended. The presence of missed cleavages in tryptic peptides represents a challenge in quantitative MS-based proteomics, which uses peptides as surrogates for their parent proteins [10]. Under optimal conditions, peptides should be stoichiometric with the parent protein to enable accurate quantitation. However, if a protein is digested into multiple overlapping peptides, the specific signal is attenuated and in consequence the quantification becomes underestimated.

There are also side reactions of trypsin known, for example its transpeptidase activity, which yields the addition of a single amino acid (Arg or Lys) to peptides in the reaction mixture. There are also dipeptides added or two longer peptides are combined together. The additions have been described for both N- and C-termini of peptides [7]. However, the incidence of the modified peptides is much lower compared to their unmodified counterparts.

## 3. Trypsin Forms

Wilhelm Kühne, who discovered trypsin and coined its name in 1876, noticed that the enzyme was produced as an inactive zymogen (trypsinogen) in pancreatic cells [11]. In the 1930s, Moses Kunitz and John H. Northrop elaborated procedures to isolate both trypsinogen and trypsin in crystalline forms, which largely contributed to the development of enzymology [12]. Trypsinogen is stable at acidic pH (2–4). In a neutral or alkaline solution, it is activated by a limited proteolysis catalyzed by either duodenal enteropeptidase (enterokinase) or trypsin itself [4]. Thus, in the latter case, an autocatalytic process occurs. The complete amino acid sequence of bovine trypsinogen was deduced from peptide sequencing experiments by several independent groups in the 1960s [13,14] with later corrections at ambiguous positions (some Asn/Asp and Gln/Glu were not distinguished in early studies because of limitations of the sequencing methods used at that time). In addition to the dominant cationic trypsinogen, a minor anionic trypsinogen is produced in bovine pancreas [15]. The UniProt accession numbers are P00760 (TRY1_BOVIN) and Q29463 (TRY2_BOVIN), respectively; the corresponding mature trypsin sequences share 72% identity.

**Figure 1:**
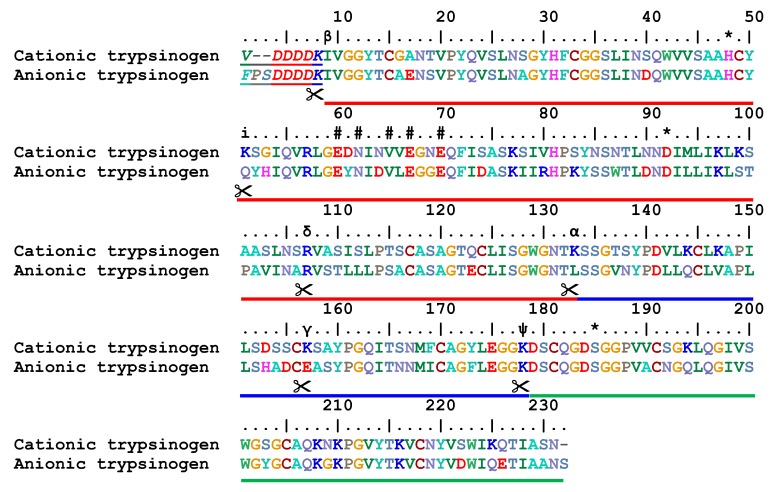
An alignment of the cationic and anionic bovine trypsinogen sequences. Italic letters and a thin underlining at the beginning of the sequences highlight the activation peptides, which are cleaved off during the trypsinogen conversion to mature trypsin. In the ruler line, asterisks (*) indicate active-site residues (the charge relay system, also catalytic triad) and hash (#) symbols mark calcium-binding residues. Greek alphabet letters indicate the cleavage sites in the active single-chain β-trypsin (223 residues), which yield the other trypsin proteoforms (α, γ, δ, and ψ) upon autolysis. The small-case letter ‘i’ denotes the cleavage site leading to an inactive trypsin variant. All above mentioned cleavage sites are additionally highlighted by a symbol of scissors. Thick red, blue, and green lines indicate individual ψ-trypsin chains. The alignment was made using BioEdit 7.2.5 with trypsinogen sequences obtained from the UniProt database. The accession numbers are P00760 (TRY1_BOVIN, cationic) and Q29463 (TRY2_BOVIN, anionic). The numbering refers to anionic trypsinogen. For cationic trypsinogen, it is shifted towards the canonical numbering by two units up because of the alignment.

The cationic trypsinogen sequence spans the length of 229 amino acids (Figure 1). Altogether, there are six disulfide bonds in the molecule connecting the following cysteine residues: 13 and 143, 31 and 47, 115 and 216, 122 and 189, 154 and 168 plus 179 and 203 [14]. By the removal of the N-terminal hexapeptide VDDDDK, the single chain β-trypsin is formed as the predominant product of trypsinogen activation. Trypsin autolysis with a cleavage between Lys-131 and Ser-132 (numbered according to the trypsinogen convention) produces α-trypsin, which has two chains connected by disulfide bonds) [4]. A further cleavage at the bond Lys-176−Asp-177 yields ψ-trypsin with three discrete chains [16]. Another known active trypsin forms include for example the two chain δ-trypsin [17,18,19] and γ-trypsin [20] with chain splits at the bonds Arg-105−Val-106 and Lys-155−Ser-156, respectively. On the contrary, a cleavage between Lys-49 and Ser-50 has been shown to inactivate the enzyme [18]. More degraded autolysis products are considered inactive [4]. Bovine trypsin is characterized by a molar absorption coefficient ε_280_ of 40,000 mol·L^−1^·cm^−1^ [21] resulting from the presence of 4 Trp, 10 Tyr, and 12 Cys residues (oxidized to disulfides in the protein) in the sequence. A certain degree of variability in these numbers can be found for trypsin enzymes from different mammalian or other sources of origin [4]. All mentioned variants are often called “trypsin isoforms” in the literature but this is not satisfactory. According to the International Union of Pure and Applied Chemistry (IUPAC), the term isoform refers only to genetic differences and not to a variation at the protein level. Hence, the real isoforms are e.g., the cationic and anionic trypsin. To solve this terminological confusion, the term “proteoform” has recently been introduced, which covers all molecular forms encoded by a single gene, including changes due to genetic variations, alternatively spliced transcripts and posttranslational modifications [22].

## 4. Pseudotrypsin Purification from the Autolyzate

In the original report from 1969, ψ-trypsin was purified from an autolyzate of bovine trypsin using isocratic ion-exchange chromatography [16]. The autodigestion proceeded at pH 8.0 and 25 °C in the presence of calcium ions for up to 6.5 h. Then *N*^a^-*p*-tosyl-L-lysine-chloromethyl ketone (TLCK), which is an irreversible trypsin inhibitor, was added at pH 7.0 to abolish any detectable activity. After a removal of low-molecular weight compounds from the protein by gel permeation chromatography (1 mM HCl as a mobile phase) and the subsequent lyophilization, the TLCK-treated autolyzate was separated on an Sulfoethyl (SE)-Sephadex C-50 column (1 × 48 cm) in 100 mM Tris-HCl, pH 7.1, containing 20 mM CaCl_2_. This procedure had previously been developed for a reliable resolving α- and β-trypsin [23]. In this arrangement, ψ-trypsin was eluted prior to the elution of α- and finally β-trypsin. It was found not to contain an alkylation resulting from the TLCK treatment, which was elucidated by its decreased activity towards trypsin substrates [16]. In a similar way, ψ-trypsin was purified on SE-Sephadex C-50 in several other studies. Some differences appeared in the column length, e.g., 1.5 × 150 cm [24], or the authors optimized the composition of the mobile phase by varying sample loading, pH, flow rate and concentration of NaCl [25].

**Figure 2:**
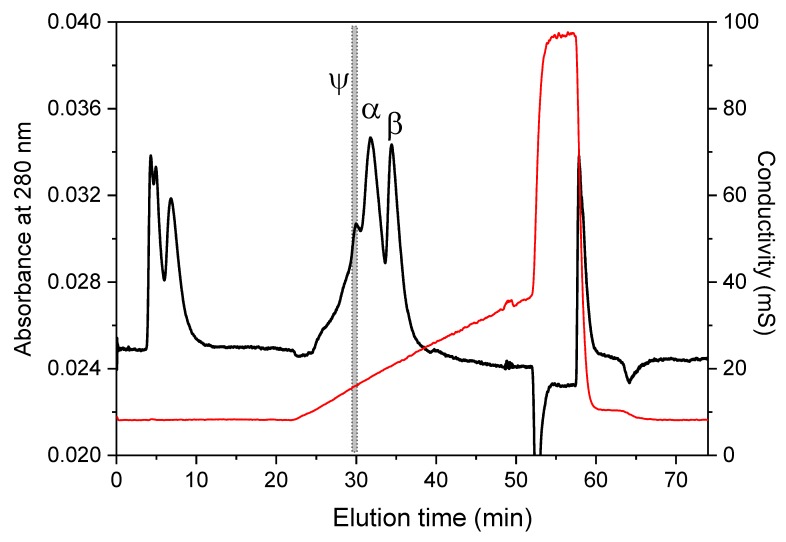
Ion-exchange chromatography of trypsin autolyzate. The depicted separation was performed on a Uno S12 column (15 × 68 mm; Bio-Rad, Hercules, CA, USA) using a linear gradient of NaCl (0–1 M) in 100 mM Tris-HCl, pH 7.1, containing 10 mM CaCl_2_. The gray box indicates a time window, in which the respective ψ-trypsin fraction was collected. The black line shows absorbance at 280 nm, the red line refers to conductivity of the eluate.

In our laboratory, we used a HEMA-BIO 1000 SB column (0.75 × 25 cm) in a medium-pressure protein liquid chromatography to separate trypsin autolyzate components [8]. The flow rate was adjusted to 2 mL·min^−1^ and the whole time window to resolve isocratically ψ-, α-, and β-trypsin (in the given order of elution times) at pH 7.1 was 45 min long. We have recently replaced the HEMA-BIO 1000 SB column by a Uno S12 column (15 × 68 mm), which allowed reducing the separation time, at the expense of resolution, but still yielded a pure ψ-trypsin (Figure 2). In this case, however, the use of a gradient elution was necessary—the buffer B contained 1 M NaCl (Perutka et al., unpublished results). In the 1970s, a French group, which studied kinetic properties of ψ-trypsin at that time, developed a convenient purification method based on affinity chromatography [26,27]. The yield was 15–20 % of the initial fully active trypsin. To prepare the affinity column, a trypsin inhibitor from egg-white chicken ovomucoid was attached to aminoethyl-cellulose using glutaraldehyde as a coupling reagent. The equilibration buffer for separation runs was 0.1 M Tris-HCl, containing 50 mM CaCl_2_, pH 7.1. ψ-Trypsin appeared already in the flow-through fraction (just after the void volume). Thus, it was necessary to include a size-exclusion chromatographic step prior to the affinity separation in order to remove low-molecular-weight contaminants such as peptides. In contrast, the proteoforms α and β remained bound and could be eluted (as an unresolved mixture) by applying an acidic elution buffer of pH 2.3 [27]. This low affinity of ψ-trypsin is in agreement with results on the formation of its complex with pancreatic trypsin inhibitor, where the corresponding dissociation constant was increased by five orders of magnitude compared to that for a mixture of α- and β-trypsin [28].

## 5. Molecular Properties and Structure of Trypsin and Pseudotrypsin

Accurate experimental molecular mass values of bovine ψ-trypsin were first determined by electrospray ionization (ESI)-MS [29,30]. For β-trypsin, α-trypsin, and ψ-trypsin, the following average numbers (relative monoisotopic molecular masses) were obtained in the given order: 23296, 23310, and 23325 [29] or 23294, 23312 and 23328 [30]. These numbers are in accordance with sequence-based calculated values of 23293, 23311, and 23329, respectively [30], which reflect a relative mass difference of 18 resulting from each consecutive autolytic cleavage. For ψ-trypsin, the most recent data are 23332 ± 4 (MALDI-MS) and 23330 ± 0.1 (ESI-MS) [8]. Thus, based on an accurate molecular mass determination, the purity of ψ-trypsin preparations can easily be evaluated, also to rule out the presence of a chymotrypsin contamination coming from the original trypsin material.

Isoelectric points of proteins can be estimated from their amino acid sequences for example using the software tool ProtParam (https://web.expasy.org/cgi-bin/protparam/). A theoretical p*I* value of the cationic bovine trypsin (Uniprot accession number P00760, positions 24–246) is 8.69. Similarly, for porcine trypsin (Uniprot accession number P00761, positions 9–231), the result is 8.26. Interestingly, higher experimental values of 10.0/10.5 [31,32] were published for the bovine enzyme and 10.2/10.8 for the porcine enzyme [33,34]. For ψ-trypsin, no experimental data on p*I* are available to our knowledge, but similar values could be expected. The anionic bovine trypsin (Uniprot accession number Q29463, positions 24–247) has a theoretical p*I* value of 4.90, which agrees well with an experimental result [35].

**Figure 3:**
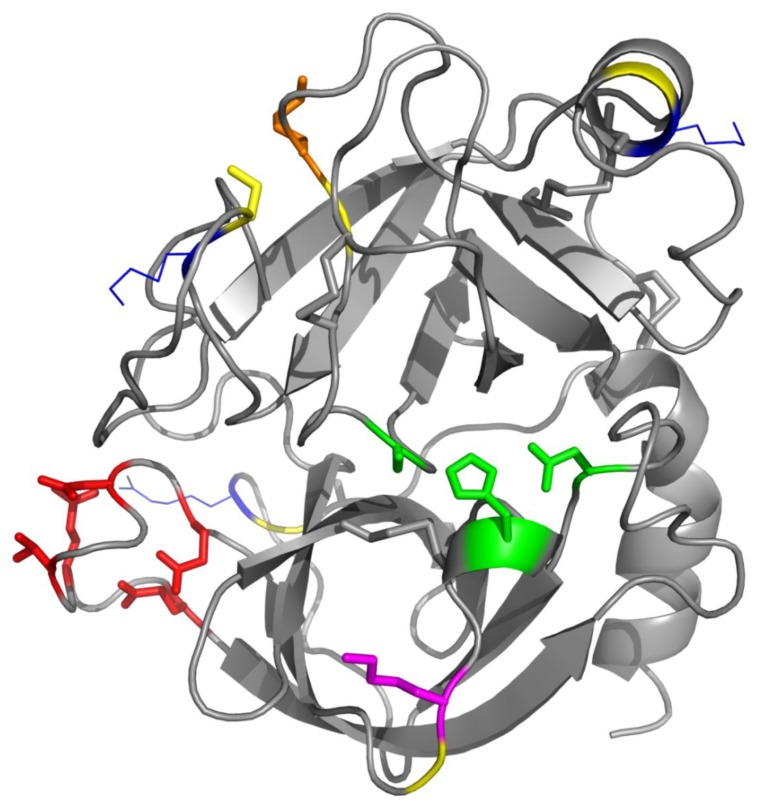
The crystal structure of bovine β-trypsin. This overall view was made in PyMOL 1.3 using a pdb formatted file (accession number 1AQ7 [41]) downloaded from the PDB database (http://www.rcsb.org/pdb). Trypsin fold comprises two β-barrels. The active site with the catalytic triad of amino acids is located between them (in the center of this figure) and highlighted in green. Red color on the bottom left shows the calcium-binding residues. The KD bond, which is cleaved to generate ψ-trypsin is expressed as an orange side chain (Lys-176) at a yellow backbone segment (Asp-177). Blue-line side chains at other yellow segments indicate the presence of the basic residues, where the β-trypsin polypeptide chain is cleaved to produce α-trypsin (Lys-131, above the calcium site and not far away from Lys-176 in this projection), γ-trypsin (Lys-155, top right, in the helix), and δ-trypsin (Arg-105, behind the calcium site). Finally, magenta color at the bottom indicates Lys-49, which is disconnected from Ser-50 to yield the inactive autolytic form.

Chymotrypsin and trypsin were among first proteins with experimentally determined spatial structures. The crystal structure of bovine chymotrypsin appeared already in 1967 [36] and was refined later on [37]. At that time, a similarity in the three-dimensional folding of trypsin and chymotrypsin could be assumed because of the amino acid sequence homology and matching positions of disulfide bonds. The crystal structures of bovine β-trypsin and trypsinogen were solved in the 1970s [38,39,40]. Figure 3 shows a view of the trypsin structure (PDB accession code 1AQ7 [41]) visualized using PyMol 1.3. Trypsin is a globular protein. Its overall fold comprises two six-stranded Greek-key β-barrels [42]. The active site with the catalytic triad of amino acids is located between the two barrels. His-57 and Asp-102 belong to the N-terminal barrel whereas Ser-195 originates from the C-terminal barrel (this numbering is according to the chymotrypsinogen convention, see in Walsh and Neurath) [13]. Helices represent only minor secondary structure components, for example at the C-terminus. The enzyme contains a calcium ion, which is important for activity. Its coordination chemistry involves several residues from the calcium-binding loop [38]. The calcium ion interacts with the side-chain oxygens (in the trypsinogen numbering) of Glu-58, Glu-65 (this one via a coordinated water molecule), and Glu-68 plus the carbonyl oxygens of Asn-60 and Val-63. No crystal structure of ψ-trypsin has been solved up to now. As this proteoform contains two chain splits (between: 1. Lys-131 and Ser-132, 2. Lys 176 and Asp-177; according to the trypsinogen numbering), the whole molecule is loosened somehow in comparison to that of β-trypsin. The bond Lys-176−Asp-177 is located close to the anionic binding site (i.e., specificity site: Asp-177, which is the position 189 when expressed in the chymotrypsinogen numbering). Upon the autolytic splitting, the binding site arrangement is disconnected. In consequence, the affinity for polypeptide trypsin substrates is lowered and the cleavage specificity broadened [8,24]. This structural alteration does not prevent from binding of a pancreatic trypsin inhibitor; only the dissociation constant of the enzyme-inhibitor complex is increased [24,28]. ψ-Trypsin still keeps a certain level of specificity, which is based on hydrophobic interactions, as confirmed using synthetic ester substrates [27].

After trypsinogen activation, the new N-terminal residue (Ile-16, in chymotrypsinogen numbering) inserts into a cleft, where it establishes an ion pair (via α-amino group) with Asp-194 next to the catalytic serine. This results in a conformational rearrangement. The amino group of Gly-193 moves into a position, which completes the oxyanion hole at the active site [2]. The hole is formed by the trypsin amide hydrogens of Gly-193 and Ser-195 in favor of stabilization of the developing negative charge on the carbonyl oxygen atom of the cleaved substrates.

## 6. Pseudotrypsin Activity with Artificial Substrates (Enzyme Kinetics)

Compared to α-trypsin and β-trypsin, the overall structural change resulting from the additional intrachain split in ψ-trypsin yields differences in the activity and specificity. This was evaluated already in the 1960s and 1970s by measuring kinetic parameters for low-molecular-weight substrates (Table 1). Smith and Shaw [16] recognized during the chromatographic purification that ψ-trypsin did not show any measurable activity with *N*^α^-benzoyl-d,l-arginine-4-nitroanilide (Bz-Arg-pNA) as a substrate under experimental conditions optimized for α-trypsin (i.e., no amidase activity). Nevertheless, they could demonstrate a hydrolytic activity of ψ-trypsin by detecting a stoichiometric incorporation of [^14^C] diisopropyl fluorophosphate at the active site, which is typical for serine proteases or esterases in general. However, the rate of ^14^C incorporation was very slow, which indicated a decreased reactivity of ψ-trypsin compared to α-trypsin. Similarly, a slow conversion of the active site titrant *p*-nitrophenyl-*p*′-guanidinobenzoate (NPGB) was observed. Comparative kinetic data measured with several artificial ester substrates are shown in Table 1. For example, the affinity of ψ-trypsin for the cationic *N*^α^-benzoyl-l-arginine ethyl ester (Bz-Arg-OEt) represented by the determined Michaelis constant value is lower by three orders of magnitude than that of α-trypsin [16]. Such a big difference in affinity was observed also for carboxybenzyl-L-lysine esters or *N*^α^-*p*-tosyl-l-arginine methyl ester [27]. Not only the affinity, but also the activity (catalytic constant) is often largely different. A typical chymotrypsin substrate *N*^α^-acetyl-l-tyrosine ethyl ester (Ac-Tyr-OEt) is hydrolyzed by α-trypsin almost 3000 times faster than by ψ-trypsin (Table 1). At the same time, the respective *K*_m_ values are comparable as the binding of this neutral substrate is not affected so much by the disconnection of Asp-177 (the specificity site) in ψ-trypsin [16]. As a consequence of the reduced affinity and activity towards cationic substrates, the efficiency constant values *k*_cat_/*K*_m_ are decreased by many orders of magnitude (up to 6) for ψ-trypsin [16,27].

Benzamidine has been shown a potent competitive inhibitor of trypsin. It is approximately of the same size as the side chain of lysine and arginine and contains both a positively charged group and hydrophobic moiety in its structure. The *K*_i_ value of benzamidine for the reaction of trypsin with Bz-Arg-pNA is 1.8 × 10^−5^ mol·L^−1^ [4]. In contrast, the binding of the inhibitor to ψ-trypsin is characterized by an increased *K*_i_ value 3.7 × 10^−2^ mol·L^−1^, which is in agreement with the *K*_m_ value for the neutral (and thus non-specific) substrate Ac-Tyr-OEt (Table 1). The irreversible trypsin inhibitor TLCK does not inactivate ψ-trypsin at all [16]. Further evidence of the reduced ψ-trypsin affinity to positively charged ligands was observed with a basic pancreatic trypsin inhibitor. The second-order rate constant of the association is decreased by a factor of 16 when ψ-trypsin is used instead of α- and β-trypsin and the dissociation constant is increased by a factor of 1.5 × 10^5^ from 6 × 10^−14^ mol·L^−1^ (a quasi-irreversible binding in the case of trypsin) to 9 × 10^−9^ mol·L^−1^. This value is similar to that of chymotrypsin, which also associates with this inhibitor but lacks the trypsin specificity site [28]. When the disulfide bond Cys-179−Cys-203 in trypsin is selectively reduced and the emerged cysteines subsequently carboxymethylated, the resulting enzyme derivative still binds the inhibitor efficiently. Interestingly, the observed kinetic parameters of the association and dissociation correspond to those determined for ψ-trypsin [28].

**Scheme 1:**
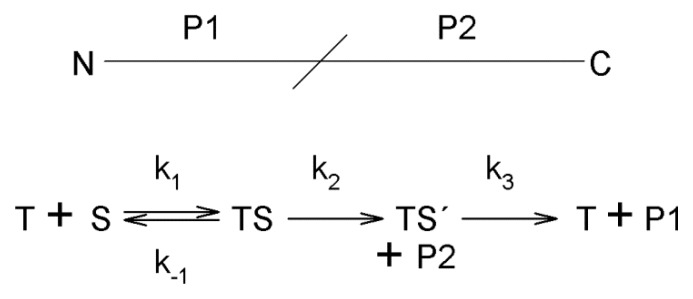
The reaction mechanism of trypsin. A protein substrate (S) with the indicated N- and C-termini and the cleavage site is cleaved by trypsin (T) into two large peptides or polypeptides P1 and P2. TS stands for a Michaelis trypsin-substrate complex, TS’ represent an acyl-enzyme intermediate. The reaction rate constants are as follows: *k*_1_—enzyme-substrate association rate constant; *k*_−1_—enzyme-substrate dissociation rate constant; *k*_2_—enzyme-substrate acylation rate constant; *k*_3_—enzyme-substrate deacylation rate constant; *k*_cat_ = *k*_2_*k*_3_/(*k*_2_ + *k*_3_). This scheme has been adapted from the reference [27].

The reaction mechanism of trypsin is illustrated in Scheme 1 (based on that in [27]) Acylation and deacylation rate constants (*k*_2_ and *k*_3_, respectively) were measured with the active site titrant NPGB and pure bovine α-, β-, and ψ-trypsin preparations [27]. Whereas the acylation rate was found to be 1000 slower for ψ-trypsin than for the other proteoforms at optimum pH, the deacylation rates were rather comparable. A similar difference in the acylation rate was observed with *p*-acetoxyphenylguanidine *p*-toluenesulfonate. This compound belongs to “inverse substrates” as it contains the specific cationic center within the leaving group instead of the acyl moiety [44]. Measurements with different carboxybenzyl-l-lysine esters (cationic i.e., specific trypsin substrates) indicated that acylation is largely the rate-limiting step for ψ-trypsin contrary to deacetylation as it appears in the case of α-/β-trypsin [27,45]. For neutral (nonspecific) substrates, acylation is rate limiting also for the major proteoforms. The *k*_cat_ values for ψ-trypsin were found sensitive to the substrate leaving group in contrast to those observed for α-trypsin, which shows that the catalysis by this form is not completely nonspecific and hydrophobic binding subsites are preserved.

## 7. Cleavage Specificity towards Peptides and Proteins

Both major forms of trypsin (α and β) prefer Arg-X sites to Lys-X sites during the hydrolysis of synthetic as well as polypeptide substrates at an optimal pH of 8–9. The preference ratio is even more pronounced at higher pH values (>pH 10) because the ε-amino group of lysine is largely discharged under these conditions and becomes less attractive [4]. The cleavage specificity of ψ-trypsin towards peptide bonds was first analyzed with two peptide substrates: a mixture of bovine and porcine glucagon and a heptapeptide fragment of the B chain of insulin with a sequence of GFFYTPK [24]. The heptapeptide was selected because of the content of aromatic residues to evaluate whether ψ-trypsin shows a preference similar to that of chymotrypsin. The glucagon sequence contains three canonical trypsin cleavage sites (Lys-12, Arg-17, and Arg-18). In a parallel work, the same group demonstrated that pure α- and β-trypsin preparations did not show any effect on the insulin-derived heptapeptide and carboxy sites of aromatic amino acid residues in glucagon [46]. Conversely to the effect of α-chymotrypsin used as a 1% model contamination in a commercial trypsin preparation (control digest), no effect of ψ-trypsin on the heptapeptide was observed. The digestion of glucagon was performed in 0.1 ammonium carbonate containing 20 mM CaCl_2_ at pH 8.0 and ψ-trypsin generated the same fragments as it had been observed in the parallel study with α- and β-trypsin. However, the yield of these fragments was lower than those produced by the major forms, indicating its decreased affinity to polypeptides. However, ψ-trypsin showed an additional ability to cleave bonds adjacent to the aromatic amino acids Phe and Trp [24].

Dyčka et al. [8] performed overnight in-gel digestions of six standard proteins with monomer molecular masses of 12–95 kDa (cytochrome *c*, lysozyme, myoglobin, glucose oxidase, serum albumin, and glycogen phosphorylase) using ψ-trypsin, non-fractionated trypsin (treated by *N*-*p*-tosyl-l-phenylalanine chloromethyl ketone—TPCK—to inactivate a possible chymotrypsin contamination) and chymotrypsin to obtain a more complex view of the cleavage specificity. The numbers of sequence-matched peptides with the respective sequence coverage values from nLC-MALDI-MS/MS were similar for ψ-trypsin and trypsin. A majority of the registered ψ-trypsin cleavage sites (77 %) were produced by its action upon the C-termini of Arg and Lys residues (in the case of trypsin, it was 86 %). Additional cleavages appeared particularly after Phe and Tyr residues, which confirmed the previous data obtained with glucagon [24]. Interestingly, ψ-trypsin provided 1.5-fold higher number of peptides containing missed cleavage (Arg and Lys) sites, which probably results from the lower substrate binding ability of this proteoform [8].

## 8. The Use of Pseudotrypsin for Protein Identification in Proteomics

At the present time, ψ-trypsin is not commonly used in biochemistry and proteomics as it is not commercially available and its purification takes a few days with a low yield of pure protein at the end. From 200 mg of bovine trypsin as a starting material [8], only milligrams of the final product could be obtained. The applicability of ψ-trypsin has a potential in protein identification experiments involving ESI- or MALDI-MS/MS as it generates more peptides with missed cleavage sites than native trypsin and also nonspecific peptides terminated mainly by Phe and Tyr residues (resembling partially the chymotrypsin mode of action). In consequence, higher sequence coverage values and increased number of matched peptides can be achieved. Interestingly, pseudotrypsin has been hypothesized to cause the cleavage of a Cys–Gly bond in NDRG1 protein [47]. Another possibility of application resides in studying posttranslational modifications. Such a modification may under a common routine interfere with trypsin digestion, for example when a phosphorylation occurs close to an arginine or lysine, and would then require selecting of another protease [48]. On the other hand, for its broader cleavage specificity, ψ-trypsin is not recommendable for mass spectrometry-based protein quantification experiments [49] because of the possible distribution of the same predicted canonical cleavage site in more peptides (multiple cleavage products due to missed cleavages or additional cleavage sites).

**Figure 4:**
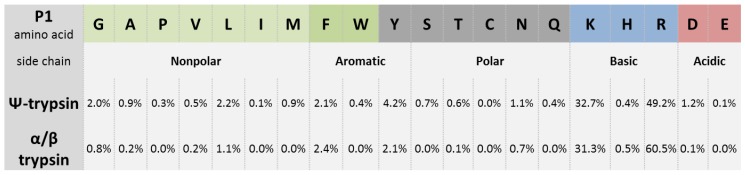
C-terminal amino acids (P1 cleavage site) of peptides in ψ-tryptic and tryptic digests. This table summarizes results obtained after in-gel digestions of barley nuclear proteins. The percentage values were averaged from data for almost 40 distinct protein fraction samples. MALDI TOF/TOF MS/MS allowed to identify 1199 and 1238 peptides, respectively. PEAKS Studio 8 software (Bioinformatics Solutions, Waterloo, ON, Canada) was used to process the MGF-formatted files by searching against a *Hordeum vulgare* protein sequence database downloaded from the National Center for Biotechnology Information, USA, at 1% peptide false discovery rate (Perutka, Z. et al.; unpublished results). Peptide sequences containing C-termini of the identified proteins were not included in the calculation.

A comparative in-gel digestion of a gel fraction of rat urine proteins resulted in 22 identifications after the use of ψ-trypsin. The same number was reached with a commercial non-fractionated trypsin, but only 17 were identified in both cases. Hence a simple combination of the two digestions provided about 20 % more identification [8]. The total number of the matched peptides was 233 and 199, respectively. The numbers of the cleaved Arg and Lys sites were comparable (around 130); in the case of Phe and Tyr site cleavages, two times more peptides were produced by ψ-trypsin. Recently, this proteoform was applied to analyze nuclear proteins after a DNase treatment of barley nuclei and sodium dodecyl sulfate polyacrylamide gel electrophoresis of the released protein material (Perutka et al., unpublished results). Peptides from the digests were analyzed by MALDI and ESI MS/MS and the results compared with parallel tryptic digestions. The identified nonspecific peptides in the ψ-tryptic digests represented 15–20 % of the total peptide number (compared to 7 % for a standard trypsin). In agreement with previous reports [8,24], peptides with C-terminal Tyr and Phe residues (and also Leu) were found in a significant percentage representation but were only minor to those resulting from the characteristic trypsin cleavage after the basic Arg and Lys residues (Figure 4). The higher number of Arg-ending over Lys-ending peptides in the MALDI-TOF/TOF MS/MS results (but not in the ESI-based results; not shown) probably reflected the fact that Arg-peptides provide more intense signals in MALDI-TOF MS [50], and thus they are preferably selected for the subsequent data-dependent fragmentation. Database searches allowed identifying novel proteins which had not previously been recognized based on standard tryptic peptides and deposited in the database UNcleProt [51]. They accounted for around 10 % of all identifications. The digestion performance of ψ-trypsin was compared relatively to that of a commercial trypsin using MALDI-TOF MS-based quantification of bovine serum albumin peptides (Perutka et al., unpublished results). Tryptic digestion was performed in a buffer made of H_2_^18^O [52]. During proteolysis, labeled standards were generated by incorporating ^18^O isotope into the carbonyl group of the nascent peptides. The ψ-tryptic digest was made in a buffered H_2_^16^O and then mixed equivolumetrically with the labeled standards. The ratios of non-labeled versus labeled peptides were calculated from the areas of isotopically resolved peaks in MALDI-TOF MS spectra. As a result, the observed overall overnight digestion performance of ψ-trypsin was found lower by around 20 %.

## 9. Concluding Remarks

Early studies identified ψ-trypsin as a proteoform resulting from trypsin autolysis. Amino acid analyses of its polypeptide chains revealed the existence of an additional split between Lys-176 and Asp-177 compared to the primary structure of α-trypsin. The enzyme can be obtained from a trypsin autolyzate by ion exchange chromatography. Purification protocols that are available utilize one of the characteristic features of ψ-trypsin: in contrast to α- or β-trypsin, it is not modified by the trypsin inactivator TLCK and shows a minimum retention on cation exchangers such as Sulfoethyl-Sephadex.

Enzyme kinetics studies with synthetic amino acid esters demonstrated a largely decreased affinity and activity towards cationic substrates. This has been elucidated by the presence of the characteristic chain split, which disconnects the specificity site (Asp-177) from the active site (Ser-183, His-46, Asp-90; all indicated according to the trypsinogen numbering convention). The reaction mechanism of ψ-trypsin with specific substrates differs from that of α- or β-trypsin in the rate limiting step. No crystal structure of ψ-trypsin has been solved yet, but the anticipated existence of the modified (‘neutral’) active site and the ability to hydrolyze tyrosine-derived ester substrates lead to cleavages characteristic of chymotrypsin. So far, only a few studies have focused on the digestion of peptides and proteins by ψ-trypsin. These studies have confirmed its preferential action on Arg and Lys residues but also at Phe and Tyr residues (which is typical for chymotrypsin) as minor cleavage sites. The produced peptides contain frequent missed cleavages because of the absent specificity and reduced affinity towards the cationic sites. However, overnight digestions of protein samples provide enough peptides for identification experiments involving nLC-MALDI or nLC-ESI MS/MS. Subsequently, combining data from tryptic and ψ-tryptic digestions has been shown to be advantageous in order to increase the number of matched peptides and sequence coverage values. Furthermore, peptides with missed cleavage sites could be beneficial for studying posttranslational modifications of proteins.

ψ-Trypsin should not be used as an equivalent substitute of trypsin or chymotrypsin and, in fact, there is no need to do it. However, as it makes preferential cleavages after basic Arg and Lys residues and has a more developed side specificity for aromatic and Leu residues, which is not expected to occur for pure major trypsin forms α and β (but may appear anyway because of their autolysis), it produces valuable complementary information. The unavailability of any commercial material represents a big obstacle for the application of ψ-trypsin in common proteomics research. On the other hand, a single-step affinity chromatographic method has already been introduced [27], which could make the preparation of the enzyme easier (after a revision and transformation with the use of modern chromatographic materials).

## Figures and Tables

**Table 1:** Kinetic data for bovine α-trypsin and ψ-trypsin with artificial substrates. This table is adapted from Smith and Shaw [16] and completed by results with non-fractionated trypsin [43]. All experiments were performed at pH 8.0 and 25 °C.

Enzyme	Substrate ^a^	*k*_cat_ (s^−1^)	*K*_m_ (mol·L^−1^)	*K*_i_ (mol·L^−1^)
α-Trypsin	Bz-Arg-OEt (0.05 M CaCl_2_)	24	2.5 × 10^−6^	-
ψ-Trypsin	Bz-Arg-OEt (0.001 M CaCl_2_)	0.19	1.1 × 10^−2^	-
ψ-Trypsin	Bz-Arg-OEt (0.05 M CaCl_2_, benzamidine)	-	-	3.7 × 10^−2^
Trypsin (non-fractionated)	Bz-Arg-OEt (0.025 M CaCl_2_)	14.6	4.3 × 10^−6^	-
α-Trypsin	Ac-Tyr-OEt (0,05 M CaCl_2_, 10% 2-propanol)	57	6.2 × 10^−2^	-
ψ-Trypsin	Ac-Tyr-OEt (0.001 M CaCl_2_, 10% 2-propanol)	0.02	3.7 × 10^−2^	-
Trypsin (non-fractionated)	Ac-Tyr-OEt (0.05 M CaCl_2_, 5% dioxane)	14.5	4.2 × 10^−2^	-
Trypsin (non-fractionated)	Bz-Arg-pNA (benzamidine) ^b^	-	-	1.8 × 10^−5^

^a^ Further information on the composition of the reaction mixture is provided in parentheses; ^b^ This reaction was performed at pH 8.15 and 15 °C.

## Abbreviations

Ac-Tyr-OEt	*N*^α^-acetyl-l-tyrosine ethyl ester
Bz-Arg-OEt	*N*^α^-benzoyl-l-arginine ethyl ester
Bz-Arg-pNA	*N*^α^-benzoyl-d,l-arginine-4-nitroanilide
ESI	electrospray ionization
IUPAC	International Union of Pure and Applied Chemistry
MALDI	matrix-assisted laser desorption/ionization
MS	mass spectrometry
MS/MS	tandem mass spectrometry
NPGB	*p*-nitrophenyl-*p*′-guanidinobenzoate
nLC	nanoflow liquid chromatography
PDB	Protein Data Bank
SE-Sephadex	Sulfoethyl-Sephadex
TLCK	*N*^α^-*p*-tosyl-l-lysine-chloromethyl ketone
TOF	time-of-flight
TPCK	*N*-*p*-tosyl-l-phenylalanine chloromethyl ketone
